# Atrial conduction velocity mapping: clinical tools, algorithms and approaches for understanding the arrhythmogenic substrate

**DOI:** 10.1007/s11517-022-02621-0

**Published:** 2022-07-22

**Authors:** Sam Coveney, Chris Cantwell, Caroline Roney

**Affiliations:** 1 Leeds Institute of Cardiac and Metabolic Medicine, University of Leeds, Leeds, UK; 2 Department of Aeronautics, Imperial College London, London, UK; 3 School of Engineering and Materials Science, Queen Mary University of London, London, UK

## Abstract

Characterizing patient-specific atrial conduction properties is important for understanding arrhythmia drivers, for predicting potential arrhythmia pathways, and for personalising treatment approaches. One metric that characterizes the health of the myocardial substrate is atrial conduction velocity, which describes the speed and direction of propagation of the electrical wavefront through the myocardium. Atrial conduction velocity mapping algorithms are under continuous development in research laboratories and in industry. In this review article, we give a broad overview of different categories of currently published methods for calculating CV, and give insight into their different advantages and disadvantages overall. We classify techniques into local, global, and inverse methods, and discuss these techniques with respect to their faithfulness to the biophysics, incorporation of uncertainty quantification, and their ability to take account of the atrial manifold.

**Graphical abstract:**

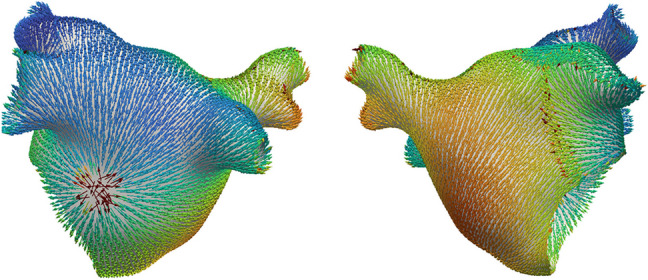

## Introduction

Atrial arrhythmias, including atrial fibrillation (AF), are a major global health problem; AF was estimated to affect 7.6 million people over 65 in the EU in 2016 [1]. To improve therapeutic strategies, which include anti-arrhythmic drug therapy and radiofrequency catheter ablation therapy, an improved understanding is needed of the factors underlying the arrhythmia of each individual patient. Characterizing patient-specific atrial conduction properties is important for understanding the arrhythmia drivers, for predicting potential arrhythmia pathways, and for personalising treatment approaches. One metric that characterizes the health of the myocardial substrate is atrial conduction velocity (CV), which describes the speed and direction of propagation of the electrical wavefront through the myocardium [2].

Atrial CV has been established to be a function of a range of functional and structural properties, including the underlying wavefront direction relative to the anisotropic fibre alignment of the tissue, the presence of atrial fibrosis, pathophysiological changes in cell-to-cell coupling and alterations in the behaviour of the sodium current [3–5]. Assessment of atrial CV in the clinic could inform the clinician on likely re-entry circuits. With more recent developments, these measurements could be used to calibrate patient-specific models to predict and identify likely conduction paths during an arrhythmia. As a final motivation for the calculation of atrial CV, areas of low CV could represent an ablation target during catheter ablation therapy.

Cardiac CV and associated mapping algorithms have been discussed in previous review articles [2, 6]. However, besides covering recent research, this review differs from these by grouping techniques into broad categories in order to offer insight into the advantages and disadvantages of different types of methods. We also assess CV analysis methods by considering the following: (i) accounting for the physics of electrical wavefront propagation; (ii) incorporation of uncertainty quantification; and (iii) accounting for the manifold nature of the atrial geometry. These characteristics are important to provide CV estimation that accounts for the physics and physiology of the heart. We then discuss the latest applications of these algorithms in the clinic, and future research directions.

### Data types, catheters and electroanatomic mapping systems

Electrogram data from electroanatomic mapping systems provide a wealth of spatiotemporal information on the progression of action potential propagation in the myocardium. Signals are obtained from multipole catheters, connected to the mapping system and inserted into the patient. By using this information to calculate electrical propagation speeds and directions at different pacing rates, the clinical electrophysiologist can gain a more comprehensive understanding of potential arrhythmia activation pathways, heterogeneity of the substrate, and the mechanism maintaining atrial fibrillation compared to looking at activation times alone.

The unipolar and bipolar electrogram modalities have been ubiquitous in clinical electrophysiology. Unipolar signals are measured between a roving electrode and a distant fixed electrode. Clean unipolar signals, in the presence of relatively simple activation patterns, enable precise timing information to be calculated. In combination with other electrodes, conduction velocity can be estimated. However, this is frequently unreliable in practice due to the impact of poor contact and far-field electrical signals. Bipolar electrograms, between two closely spaced roving electrodes, are effective at rejecting far-field activation due to their close proximity [2]. Their morphology is, however, dependent on the relative orientation of the inter-electrode axis to the direction of activation. The recently proposed *omnipolar* signals mostly overcome this direction-dependence by using three or more non-colinear signals to infer directional information about the local electric field and consequently compute a local virtual bipolar signal oriented orthogonal to the wavefront, although there are implied assumptions of locally planar conduction and they continue to be susceptible to the other issues of poor contact [7].

There are now many different catheters available for recording contact electrograms, with different electrode arrangements, inter-electrode spacing and coverage. Local multipolar mapping catheters are a relatively low-cost and widely available tool for creating local activation time or voltage maps for the cardiac chambers. These catheters typically consist of twenty unipolar electrodes spanning a diameter of 1.5–2 cm with different configurations, including a spiral (Afocus II), a circle (Lasso), and a five-spline arrangement (PentaRay) [8, 9]. Bipolar electrogram recordings are constructed from pairs of unipolar electrograms, and the amplitude and morphology are affected by the inter-electrode spacing, electrode contact and wavefront direction. Other more global electrode arrangements include the Constellation basket catheter, which consists of eight splines of eight electrodes, which allows measurement from a larger surface area of the atria, at a lower resolution [10, 11]. More recently, high-density grids of electrodes are available (HD Grid) which provide relatively stable inter-electrode distances in both directions and aids in the calculation of omnipolar signals [12]. Higher density plaque and basket (Orion) electrode arrangements are also available [13, 14]. It is important to consider the spatial and temporal resolutions associated with each of these catheters when calculating and interpreting CV [15, 16].

Electroanatomic mapping systems provide increasingly detailed analysis modules in their latest releases. The CARTO Prime module from Biosense Webster integrates their *Coherent Mapping* analysis, which includes algorithms to identify the most probable global propagation map; display CV vectors; and indicate areas of slow or no conduction [17, 18]. EnSite from Abbott includes omnipolar signal analysis for HD grid recordings, and the *LiveView Dynamic* module displays activation directions and maximum voltage maps to identify regions of wavefront collision and conduction block [19]. Lumipoint from Rhythmia, Boston Scientific, also highlights regions of interest, fractionated signals and localizes areas of slow and narrow conduction [20]. It uses the Intellamap Orion 64 electode high-density catheter to clearly identify gaps in re-do pulmonary vein isolation procedures [21]. Kodex-EPD Philips cardiac imaging and mapping system uses dielectric mapping to estimate wall thickness [22], which can be compared to CV using openEP software [23]; future iterations of the software may combine these analyses. Acutus Medical AcQMap offers high-resolution global maps, and includes a *SlowZone Locator* where multiple maps are combined to identify regions of consistently slow conduction [24, 25]. Exciting new developments across the electroanatomic mapping systems offer detailed analysis of the atrial substrate.

### Local activation time

The majority of the algorithms covered in this review article require the accurate annotation of local activation time (LAT) on each acquired electrogram. Approaches to this have been discussed previously [2], and we refer the reader to this review article for further details about many of the historical methods for LAT annotation. More recently an approach has been proposed which incorporates uncertainty quantification (UQ) for LAT annotation and once defined, how to use it in probabilistic interpolation, but this was mostly a heuristic approach [26]. We briefly discuss LAT assignment for complicated activation, such as during atrial arrhythmias, in Section 6.

### CV method categories

This review places CV calculation methods into three categories: **(1) local methods**, whereby CV is calculated using only LAT observations from an immediate spatial neighbourhood; **(2) global methods** in which a complete CV map is fitted to all LAT observations simultaneously; and **(3) inverse methods** which infer the CV field most consistent with LAT observations in a way that accounts for physics. Our aim in this review is to give insight into the overall advantages and disadvantages of these different categories of methods.

## Local methods

Local methods attempt to reconstruct the CV field at a particular position using LAT measurements in a nearby spatial neighbourhood. Examples for defining this spatial neighbourhood include the span of a catheter’s electrode configuration, a fixed distance threshold, and a Delaunay triangulation of measurement locations. A common disadvantage associated with local methods is that the resulting CV vectors can appear quite non-smooth, due to their limited ability to handle noisy measurements and the discontinuous nature of nearest-neighbour algorithms. Nonetheless, a significant advantage of these methods is that reasonable assumptions can be made about wavefront propagation in a local region even if the overall activation pattern is more complex. With high-density measurements, these methods may also enable analysis of conduction velocity heterogeneity, and identification of small areas of conduction slowing.

### Triangulation

**Fig. 1:**
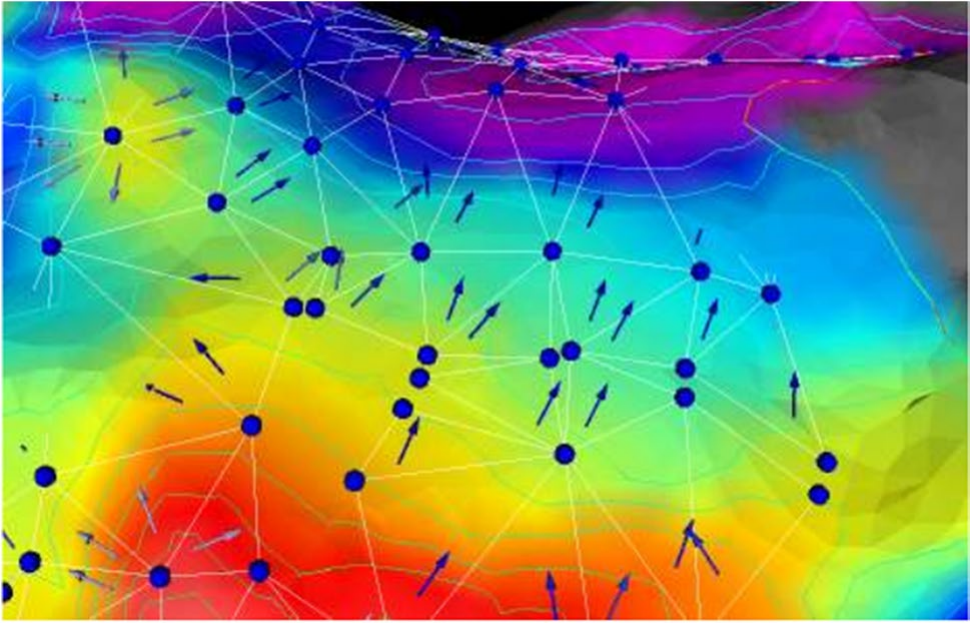
LAT measurement locations (dark blue circles) connected by a Delaunay triangles (white lines), with CV vectors shown as arrows. Figure reproduced from [31]

One of the simplest methods to calculate conduction velocity is across a triangle of points, since at least three non-collinear points are required to define the gradient vector of LAT in a 2D plane. The first triangulation method used a catheter with an equilateral triangle of electrodes to measure CV at specific catheter placements [27]. Data collected with high-density grids of electrodes can also be triangulated by considering triplets of neighbouring electrodes [28], and this method can be generalized to non-equilateral triangles [29, 30]. A Delaunay triangulation of arbitrarily positioned measurements can be defined over a manifold in order to calculate CV vectors in 3D [31–33], as shown in Fig. 1. Attempts have been made to account for geodesic distances in these methods [34].

In the references above, trigonometric formulas are used to define CV. However, all of these methods assume a planar wave propagation with constant velocity, and so a much simpler parameterization of the problem is possible by expressing LAT as a linear function of position in a 2D plane using in-plane coordinates:

1 $$T_{i} = \alpha_{0} + \alpha_{1} X_{i} + \alpha_{2} Y_{i}$$

from which the slowness vector ∇*T* = (*∂T*/*∂x*,*∂T*/*∂y*)^*T*^ = (*α*_1_,*α*_2_)^*T*^ can be immediately defined, and therefore conduction velocity defined via *CV* = ∇*T*/|∇*T*|^2^. These coefficients can be related to CV magnitude and angle of incidence via simple trigonometry [35]. Piecewise linear polynomials can also be used to calculate the gradient of a function on a triangle using values defined at vertices [36]. This parameterization shows that measurement error cannot be accounted for with these methods, since the linear model has three parameters and three measurements, therefore the fit will always have zero residuals.

### Flexible smooth functions

Fitting smooth functions *T*(**x**) such as quadratic 2D polynomials [37] and cubic 3D polynomials [38, 39] allow for CV to vary in space. Using more measurements than model unknowns also allows for data to be noisy. Fitzgerald et al. compared polynomial fits to a large amount of data against (first-order polynomial) linear fits using a subset of data in a smaller spatial region, suggesting that the latter might detect smaller-scale features better (although higher-order polynomials fit to data subsets was not studied) [40]. Higher-order polynomials require more data for fitting (e.g. at least 10 data points for a 2D cubic polynomial), thus necessitating the consideration of larger spatial neighbourhood. These fits do not consider the spatial manifold on which the data lies, but this consideration will become more important as the spatial neighbourhood increases in size. Lou et al. take an interesting approach here by first mapping the manifold surface to longitude and latitude coordinates, and fitting quadratic polynomials to these coordinates [41]. Radial basis functions (RBFs) are another flexible choice for fitting LAT fields to the data [42, 43], also suitable for application to an arbitrary configuration of measurement locations. It is possible to use RBFs with geodesic distances in order to account for the manifold.

It would be possible to alleviate some of these problems with noisy data by fitting using Regularized Least Squares (RLS) in place of ordinary least squares (OLS). For example, RBF fits will pass through the data points exactly using OLS (it is almost always the case that the basis functions are centred on each data point), but this can be overcome with RLS. This also has a Bayesian interpretation, allowing for the posterior distribution of the fitted coefficients, and therefore uncertainty on CV, to be calculated (see Appendix A).

### Catheter specific

**Fig. 2:**
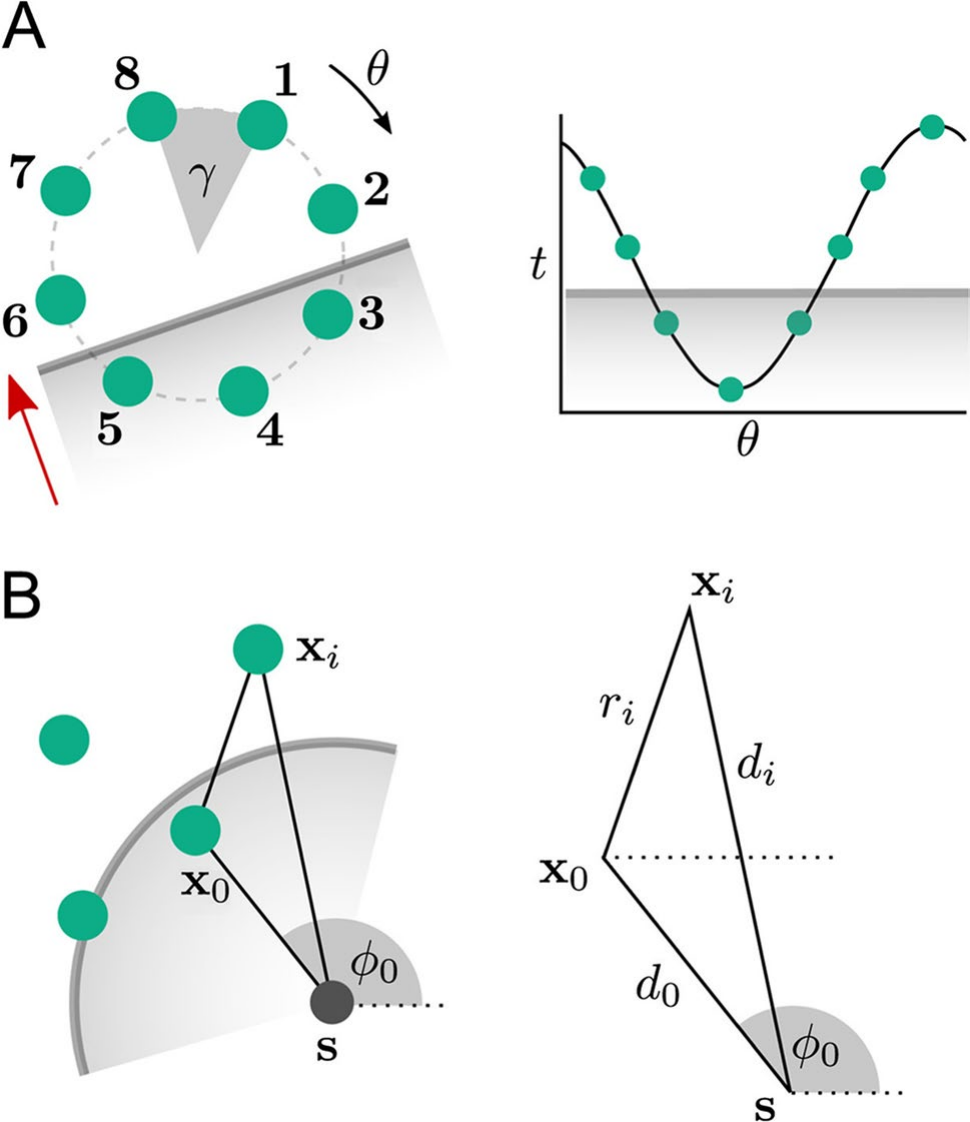
(A) The *cosine method*, (B) circular wavefront and arbitrary measurement positions. Figure reproduced from [2]

Methods for calculating CV may be derived for specific catheter configurations. The triangular catheter method of [27] is one such example. The *cosine method* makes use of data collected from a circular catheter [43–46], and assumes a plane wave with constant velocity, for which the LAT data can be fit with a cosine function, from which CV can be calculated; see Fig. 2(A). The relatively large number of measurements on the decapolar catheter should provide robustness to noise. The *cosine method* assumes a planar wave with constant velocity, so it is not clear whether there is any real advantage over simply using Eq. (1). The extension to a circular wave (see Fig. 2(B)) for specific configurations of electrodes has been shown by [47]. Linear catheters paced from one end also provide a particularly simple solution to measuring CV, since the time differences and distances along the catheter allow CV to be easily calculated, e.g. [48, 49]. This method assumes that the wavefront is perpendicular to the catheter.

Finite difference methods, operating on regular grids of data, provide another possibility. Although mostly used for optical mapping, high-density grids of electrodes are also suitable for finite difference methods [50, 51]. Finite difference methods are very susceptible to noise, and as is commonly known, for a 5-point stencil consisting of an electrode and its four nearest neighbours, central difference first derivatives do not depend on the value measured at the central electrode. This is unfortunate since this is a very important data point for estimating CV at the central electrode, and is easily accounted for by other fitting methods. Other methods using high-density grids of electrodes have also been suggested [52, 53].

### Generalized wave fitting

As mentioned in the sections above, many methods of calculating CV assume a plane wave propagation at constant CV, which produces a linear LAT surface as a function of 2D space. This raises the question of why not just fit such a LAT surface to data directly. For circular waves, a general fit to a non-specific arrangement of points is also possible. One catch is that points on a 2D manifold in 3D are not coplanar in general, although for a specific catheter placement they might be assumed to be. To generalize these methods to any arrangement of data points therefore requires projection of the data into a 2D plane.

**Fig. 3:**
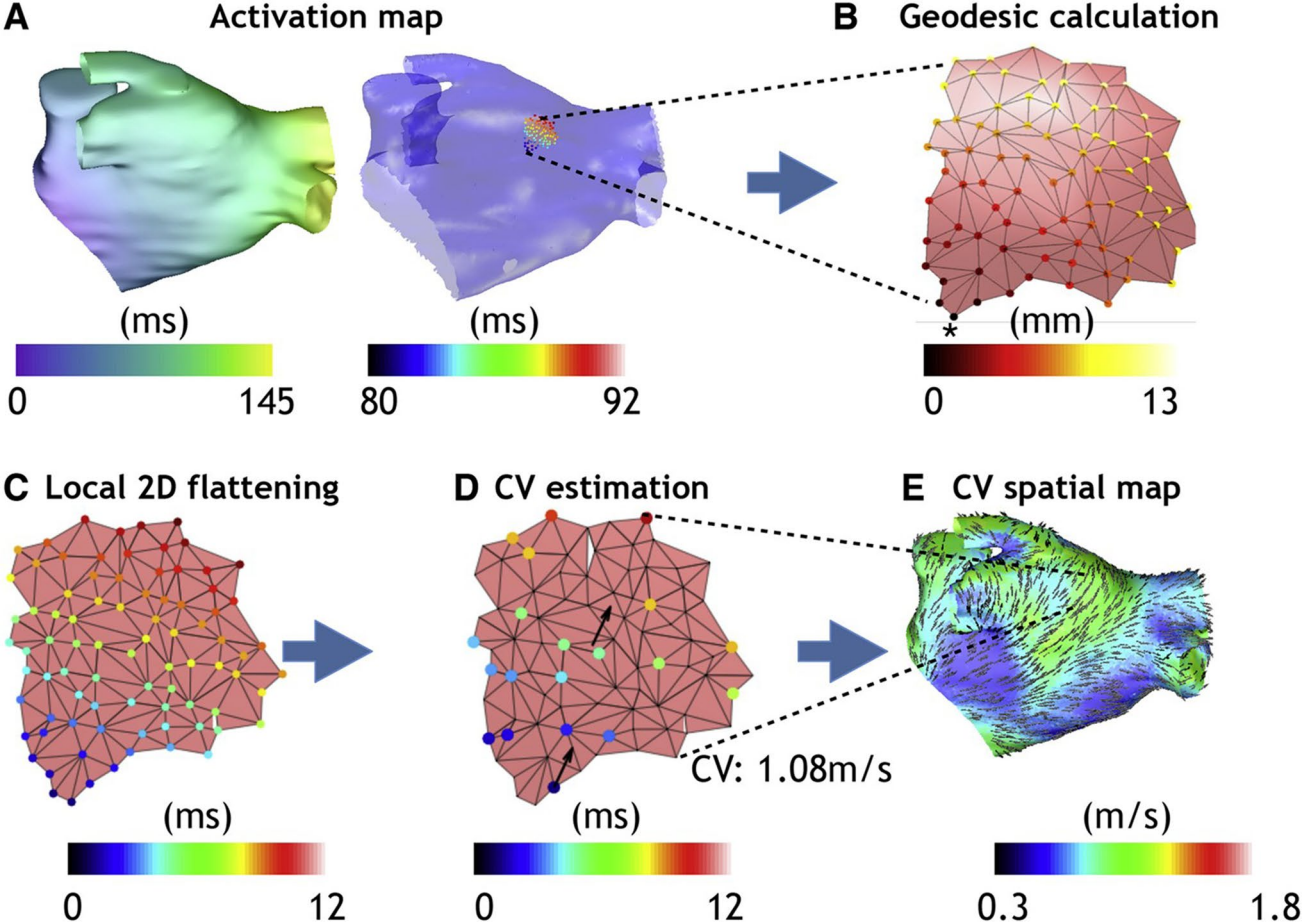
Generalized wave fitting method. A patch of mesh is projected down into 2D coordinates, and CV is calculated assuming either a planar wave or circular wave in these local coordinates. Figure reproduced from [35]

The techniques presented in [54] generalize plane wave and circular wave fitting to arbitrary configurations of points, by first projecting the points down into the 2D plane of best fit by least squares through the 3D data coordinates. The method in [35] improves upon this method by explicitly taking into account the manifold using a multi-dimensional scaling technique presented by [55]: the geodesic distances are calculated between all measurement locations on the mesh, and used to project the points into a 2D coordinate system that best preserves these inter-point distances. Either plane wave or circular wave fits can then be performed in this flattened coordinate system. See Fig. 3. It should be noted that, like many other fitting methods presented above, these methods assume homogeneous CV and therefore high-frequency variations in CV are smoothed out. However, higher-order polynomials could be fit to data in the flattened coordinate systems.

### Electric field from omnipolar signals

The omnipolar algorithm infers the electric field from a set of bipoles and expresses it in terms of a physiological coordinate frame, consisting of axes normal to the tissue, and parallel/perpendicular to the components of the wavefront that are tangential to the tissue. The algorithm works under the assumption of activation being a local planar travelling wave with constant velocity. Although derived from bipolar signals, this form of CV differs from the others discussed above in that it can be estimated without explicit annotation of the electrogram to obtain LAT.

In 1D, a travelling wave *ϕ* centred at *x*_0_ at time *t*_0_ looks the same as the wave centred at *x*_0_ + *vt* at time *t*_0_ + *t*, i.e. *ϕ*(*x*_0_,*t*_0_) = *ϕ*(*x*_0_ + *vt*,*t*_0_ + *t*). Taking the derivative with respect to time and applying the chain rule leads to

$$\begin{array}{@{}rcl@{}} 0 = \frac{d\phi(x_{0} + vt, t_{0}+t)}{dt} = \frac{\partial \phi}{\partial x} \frac{\partial x}{\partial t} + \frac{\partial \phi}{\partial t} = \frac{\partial \phi}{\partial x}v + \frac{\partial \phi}{\partial t} \end{array}$$

Moving to three dimensions and applying the relation between the electric field **E** and the extracellular potential *ϕ*, **E** = −∇*ϕ*, it can be deduced that:

$$\begin{array}{@{}rcl@{}} \mathbf{E}\cdot\mathbf{v} = \frac{\partial \phi}{\partial t}, \end{array}$$

or alternatively ${v} = (\dot {\phi } / E_{a}) {\hat {a}}$, where $\hat {\mathbf {a}}$ is the propagation direction, determined by the omnipolar fitting algorithm, and *E*_*a*_ is the component of the electric field in that direction. Since the CV from omnipolar analysis is obtained from a ratio of signals, it does not necessitate specific activation times [7]. In [56], the technique is modified to estimate the omnipolar electrogram after inter-electrogram alignment within each clique to reduce the residual angular dependency of the classical omnipolar electrogram. In addition, [56] use the ratio of standard deviations $\mathbf {v} = ([\dot {\phi }]_{SD} / [E_{a}]_{SD}) {\hat {\mathbf a}}$ rather than peak-to-peak amplitudes, with the aim of reducing the effects of noise on the estimate.

### Summary of local methods

This section has covered local fitting methods for calculating CV where a travelling wavefront is assumed. Although not applied to local methods to our knowledge, there is no reason that uncertainty quantification cannot be performed with local methods. Besides from flexible smooth functions like RBFs and polynomials, most methods assume a constant CV model for the data in the fit. This assumption will become less valid as the spatial neighbourhood becomes larger: for small regions this may be a fairly good assumption, and for large regions this may yield a low-resolution result for average CV in the region. Methods for identifying wavefront collisions have been suggested [57, 58], which may allow for calculating CV for each wavefront separately. Methods for measuring CV around re-entrant circuits have also been studied but we have not directly addressed these here (see [59–62] for examples).

## Global methods

Global methods attempt to reconstruct the LAT field on the manifold by interpolating/regressing all LAT measurements simultaneously, and CV can be derived from these global LAT maps. These methods are purely data driven and do not allow for including physics constraints (note that we categorize several methods involving a global LAT interpolation with physics constraints as ‘inverse methods’, and these are discussed in Section 4). For ease of presentation, we first discuss global methods of LAT interpolation/regression, before addressing how CV is calculated from these results.

### Radial basis functions

Radial Basis Functions (RBFs) are powerful mesh-free methods for interpolation and regression. It is almost always the case that an RBF is centred on each observation location *x*_*i*_. The basis functions then have the form *R*_*i*_(**x**) := *R*(*d*(**x**,*x*_*i*_)), depending only on the distance *d*(**x**,*x*_*i*_) and usually hyperparameters that control the RBF shape (depending on the choice of basis function). RBFs are used for LAT interpolation by both [63] and [64]. Both use ‘polyharmonic splines’, consisting of RBFs with additional polynomial terms, as follows:

2 $$f(\mathbf{x}) = {\sum}_{i = 1}^{N} \beta_{i} R(||\mathbf{x} - \mathbf{x_{i}}||) + {\sum}_{j = 1}^{M} \alpha_{j} P(\mathbf{x})$$

Both [63] and [64] used Euclidean distances, which cannot account for the manifold, and interpolating conditions, which cannot account for observation error. However, we note the following: (i) to account for the manifold, Euclidean distances can be replaced with geodesic distances and the polynomial term dropped entirely (this term is usually included to improve extrapolation, where CV estimation is undoubtedly very poor); (ii) to account for noisy observations, smoothing interpolation is possible using regularized least squares; in fact the posterior distribution can be easily obtained — see Appendix A. We mention these ideas here so that RBFs are not discounted against other published global methods that account for the manifold and for uncertainty quantification.

### Gaussian processes

**Fig. 4:**
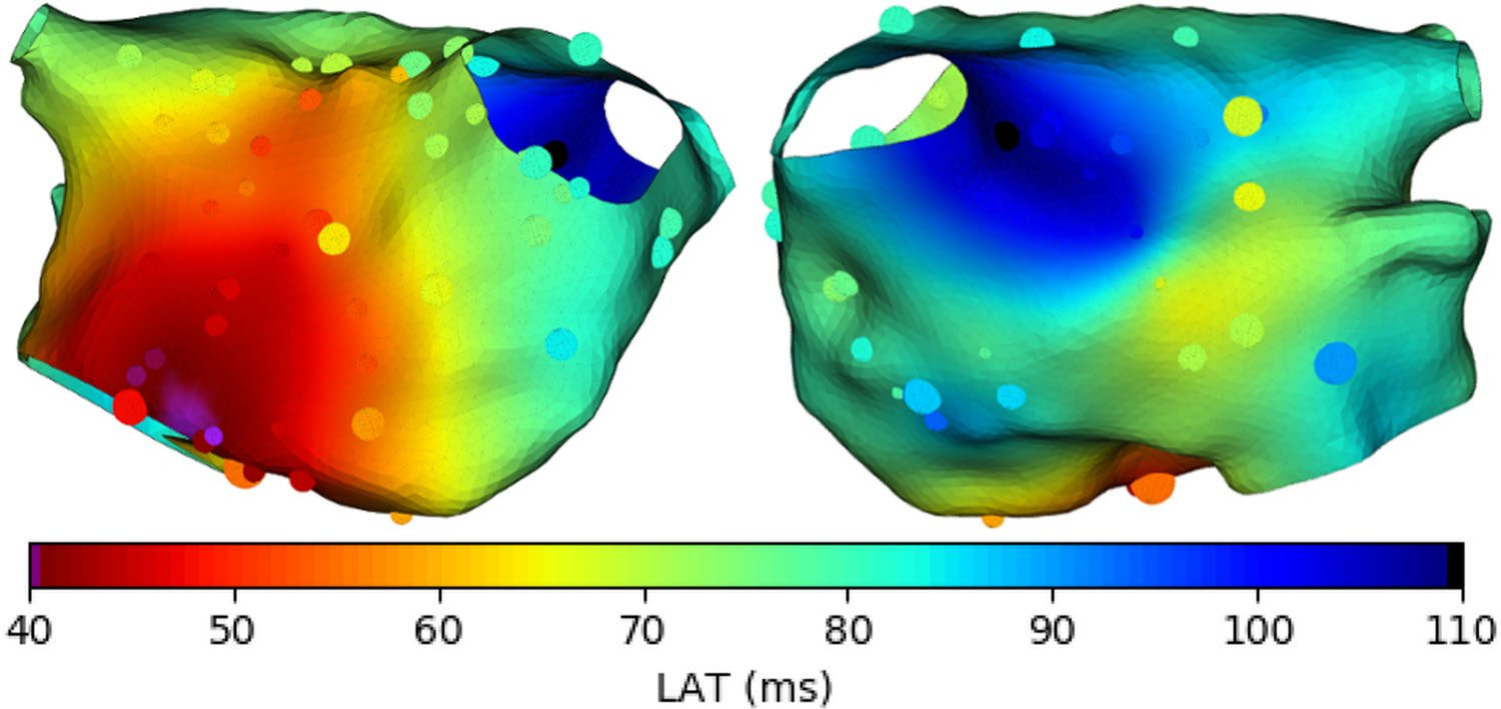
Posterior mean of LAT from the Gaussian process model of [26]. Measurement point sizes reflect measurement uncertainty. Figure reproduced from [26]

The first global method accounting for both the manifold and noisy LAT observations was given by [26]. The LAT field was modelled as:

3 $$\begin{array}{@{}rcl@{}} f(\mathbf{x}) = \beta_{0} + {\sum}_{k=1}^{n} \beta_{k} \phi_{k}(\mathbf{x}) \end{array}$$

where the basis functions *ϕ*_*k*_(**x**) are piecewise linear for every node on a triangular domain, and *β*_0_ is an intercept. This model is a Gaussian process, as the prior distribution for the probabilistic weights *β*_*k*_ is multivariate normal, with a particular form of precision matrix (inverse covariance matrix) accounting for the manifold. Although CV can be calculated from the posterior mean, which provides a good global LAT interpolation (see Fig. 4) and corresponding CV, the posterior samples of such a model are not smooth enough for calculating a posterior distribution for CV by sampling from the posterior distribution for LAT.

To alleviate this problem, [65] generalized Gaussian processes to non-Euclidean manifolds for the first time, using the following model based on theory provided in [66]:

4 $$f(\mathbf{x}) = {\sum}_{k = 1}^{M} \beta_{k} \sqrt{S(\sqrt{\lambda_{k}})} \phi_{k}(\mathbf{x})$$

where $\beta _{k} \sim \mathcal {N}(0, \sigma _{\beta }^{2})$, *S*(⋅) is the spectral density of a covariance kernel (the intercept was omitted by centering the data, but can generally be included in the model), *ϕ*_*k*_(**x**) and *λ*_*k*_ are eigenfunctions and eigenvalues of the cotangent Laplace-Beltrami operator on the domain. Note that shortly after the publication of [65], an independent derivation of the same result for Gaussian processes on manifolds appeared in [67].

### CV calculation with global methods

**Fig. 5:**
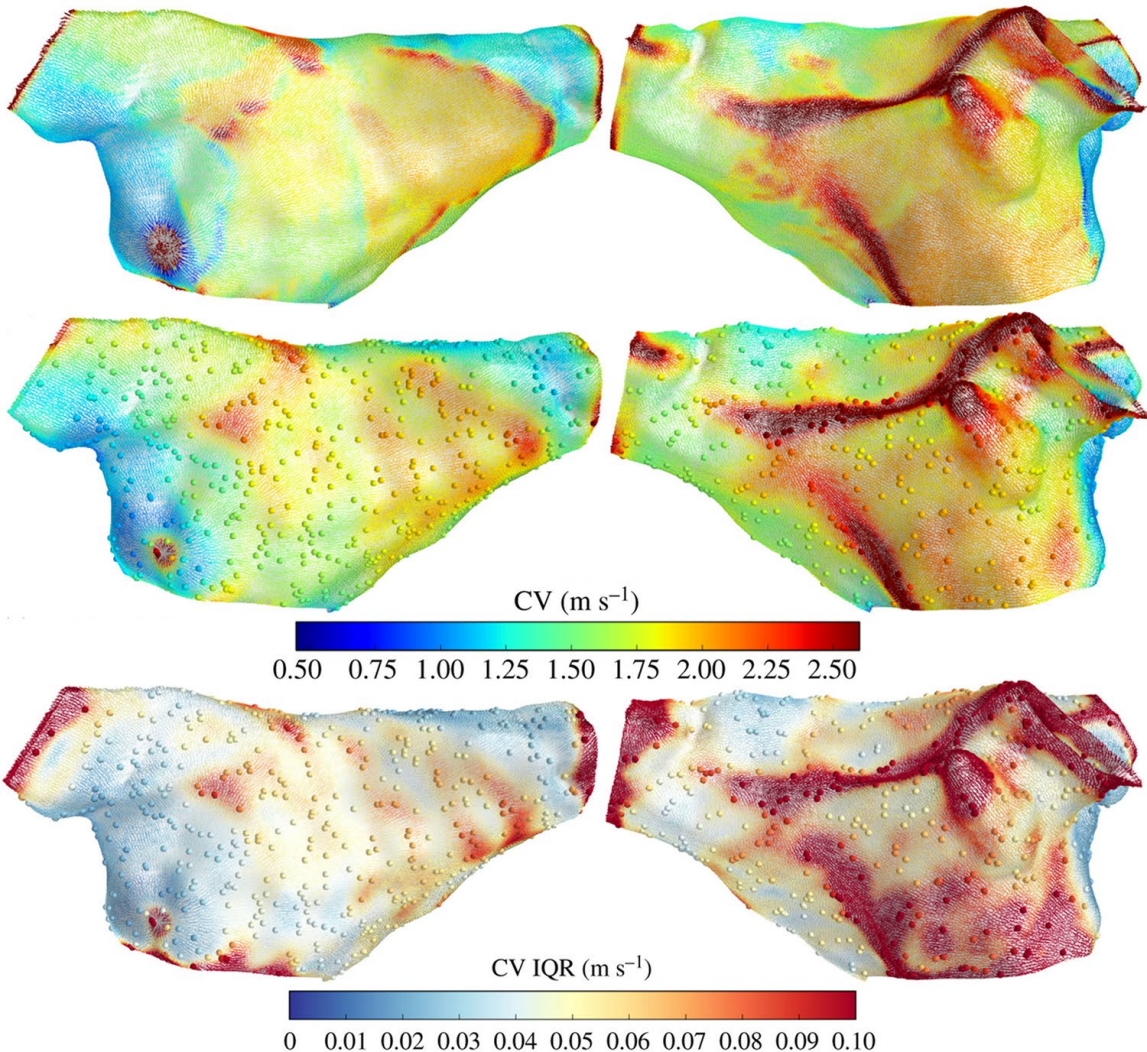
CV simulation ground truth (top) and posterior prediction and uncertainty, obtained with the Gaussian process model in [65] which generalized Gaussian processes to manifolds. Figured reproduced from [65]

Global methods obtain a LAT map, from which the spatial gradient of LAT ∇*T*(**x**), also called the ‘slowness’ vector, can be obtained and inverted *CV* = ∇*T*(**x**)/|∇*T*(**x**)|^2^. The slowness vector can be calculated on every mesh element using piecewise linear functions [36]. It is also possible to subdivide the mesh first and calculate gradients of the basis functions [65] (Fig. 5).

Isochronal methods use the distances between isochronal contours to calculate CV, effectively fitting distances between wavefronts to activation times in order to calculate CV. These results are often averaged across pairs of points on the wavefront to increase robustness, and are mostly applied to LAT maps obtained with clinical mapping system [3, 48, 68–76]. However, these methods could be applied for any global LAT interpolation technique, and it would even be possible for use them for probabilistic models by applying them to posterior samples of LAT maps. Isochronal methods utilize global LAT maps, but are similar to local methods that fit a single constant CV model over a spatial region.

### Summary of global methods

The main advantage of global methods is that all data can be accounted for simultaneously, and predictions of LAT and CV can be made everywhere. However, these models cannot guarantee physical behaviour, and for probabilistic models where the posterior mean looks reasonable, the posterior samples may not do. The global methods discussed above cannot model singularities where wavefronts collide, since they will interpolate LAT continually over these regions and the results here have no physical meaning, although this would be true of local methods fit to such regions unless the data was partitioned into different wavefronts first.

Although not used for LAT interpolation, [77] presents a generalization of spline interpolation for manifolds that makes use of Laplacian eigenfunctions weighted by the inverse of the corresponding eigenvalues. Note that the Laplacian eigenfunctions also allow various other measures of distance over a manifold to be defined, such as biharmonic distance [78]. It is possible that Laplacian-based techniques for minimizing variation of a smooth function could be utilized for LAT interpolation [79, 80]. A method to calculate a consistent set of activation times at all measurement locations was suggested by [81]. We do not know for sure what different proprietary clinical systems use for LAT interpolation, so we do not attempt to comment on these here.

## Inverse methods

Inverse methods explicitly model the diffusion/conductivity/CV field and link it to observations in a way that accounts for the physics of electrical propagation. It could be argued that some local methods also do this (e.g. fitting a plane wave of constant velocity), but these methods are restricted to the assumption of constant CV. Inverse methods model a heterogeneous CV and optimize it with respect to the data. The methods presented below are global, but we believe that the explicit modelling of CV and physics, not present in the ‘global methods’ presented above, warrants an entirely separate category. It is possible that local inverse methods will be developed in the future.

### Physics informed neural networks

**Fig. 6:**
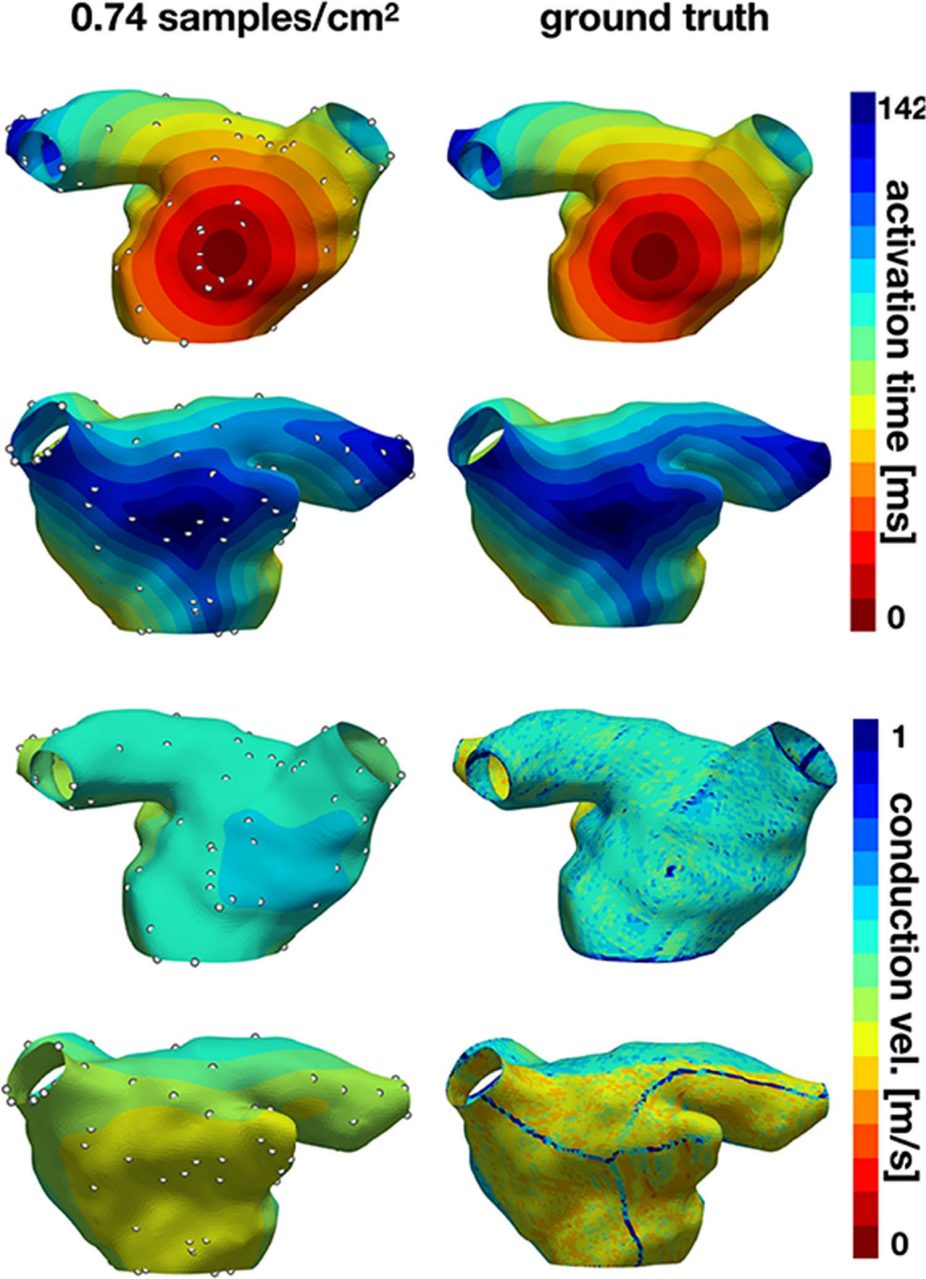
Predicted LAT maps and CV maps from a physics inspired neural network (PINN), using LAT observations at the locations indicated by white points. Figure reproduced from [82]

Physics Informed neural networks (PINNs) were introduced for simultaneous LAT interpolation and CV prediction by [82] (Fig. 6). The idea is to represent LAT and CV by separate neural networks, *T*(**x**) ≈ *NN*(**x**,*𝜃*_*T*_),*V* (**x**) ≈ *NN*(**x**,*𝜃*_*V*_), and to train the parameters of these networks using a single loss function that penalizes (i) differences between predicted and measured LAT; and (ii) residuals of the Eikonal equation *V* (*x*)||∇*T*(*x*)||− 1. Additional regularization terms are also included in the loss function. In this way, the physics of electrical wave propagation in excitable media are approximately obeyed, so the resulting solutions should be more physically realistic even though the neural networks are regressing LAT and CV using 3D Euclidean distance that do not account for the manifold. Further exciting research on PINNs that model heterogeneous diffusion fields is also underway [83]. The approach is effectively the same, but neural networks are used to model tensors representing diffusion fields and the loss function utilizes the anisotropic Eikonal equation.

[82] show that the networks can approximate discontinuities in the LAT field that occur where wavefronts collide, something that other techniques find difficult to model. However, the general performance of the method on atrial manifolds was demonstrated only for extremely simplified cases and was only compared against simple linear interpolation in 3D Euclidean space. Training such networks can also take hours [83]. Nonetheless, PINNs show great promise, and it seems reasonable to assume that further developments in the loss function and the neural network architecture might improve performance.

### Eikonal simulations

**Fig. 7:**
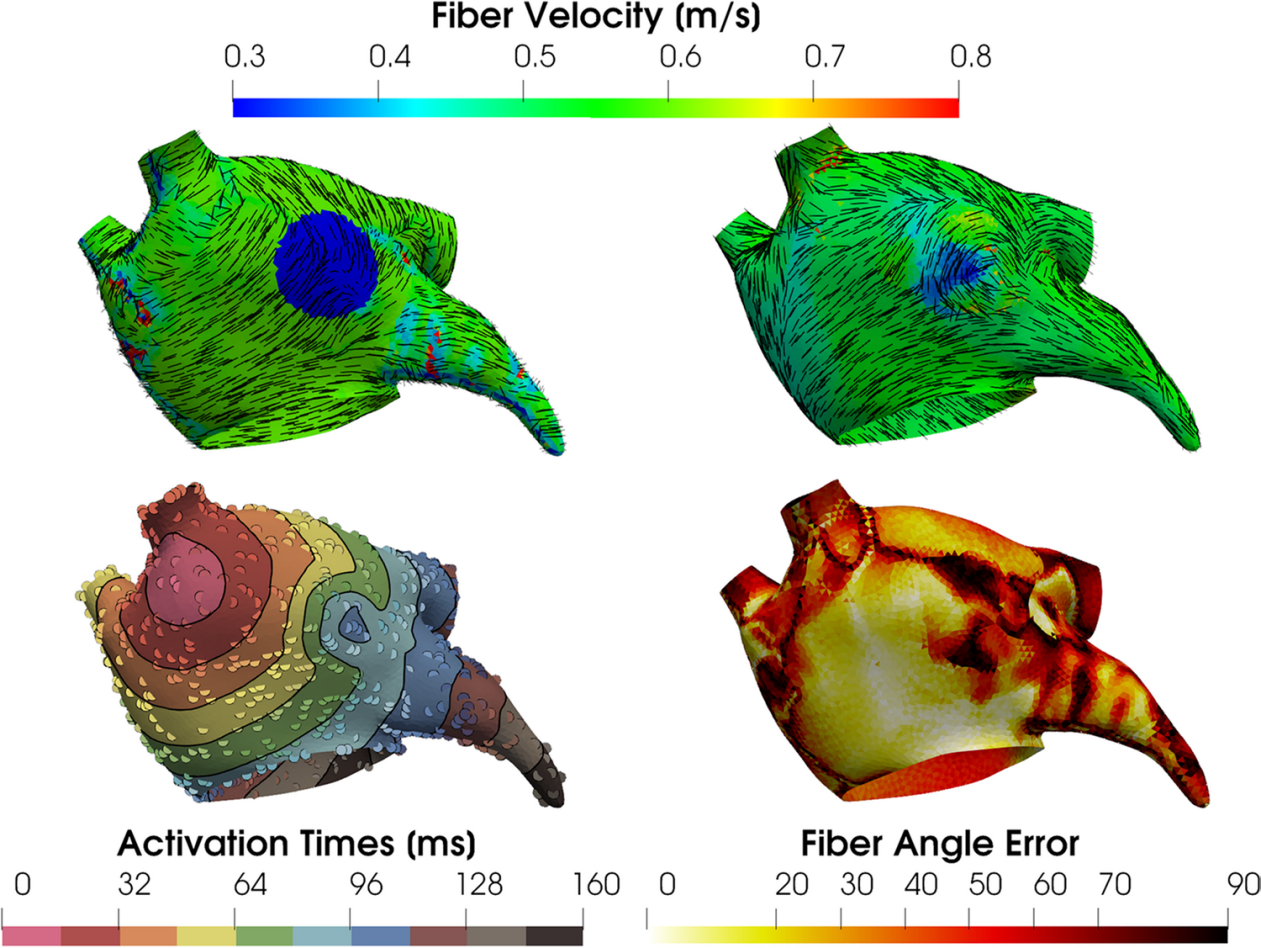
Ground truth fibres (top left) and fitted fibres inferred with *PIEMAP* (top right), along with simulation LAT and LAT observations (bottom left) and fibre angle error (bottom right). Figure reproduced from [88]

PINNs only weakly imposed the Eikonal equation via the loss function. Another approach called *PIEMAP* [84, 85] is to learn the heterogeneous diffusion field by running full anisotropic Eikonal simulations [86] in order to optimize the diffusion tensor field (Fig. 7). By comparing simulated LATs to measured LATs and by regularizing the solution in the loss function, it is possible to learn the diffusion tensor field. Similar to [83], this methodology requires that a coordinate system is first formed on the manifold, describing two orthogonal directions on each element of the mesh that vary smoothly between neighbouring elements. This may preclude the ability to learn certain fibre fields, but it may be possible to use a fibre field coordinate system based on a physiological prior [87]. Similar to PINNs, training time is much higher than local or global approaches, and is reported as around 1 hour for a high-end desktop with a GPU. A large disadvantage of PIEMAP is that the location of first activation is not learned along with the parameters, something that is not an issue for PINNs or for global methods. However, it is likely that this can be overcome by future work. Similar to PINNs, this promising research has so far only been demonstrated for fairly simplified cases.

### Summary of inverse methods

The inverse methods discussed here have enormous potential for recovering CV and diffusion tensors, since physics is imposed on the solutions via the Eikonal equation. There is a lot of recent work using deep-learning methods to solve the Eikonal equation [89–91]. These inverse methods should easily allow incorporation of LAT measurements from different stimulus positions, whereas for other approaches it is less clear how to combine this information to obtain a single estimate of CV or diffusion (a local method for learning diffusion fibre fields from multiple activation maps is given by [35]). While inclusion of uncertainty in LAT measurements via the loss functions would be fairly straightforward, posterior (prediction) uncertainty is less straightforward, e.g. the form of uncertainty quantification attempted in [82] requires training of 10^1^ − 10^2^ neural networks. These methods are also more computationally expensive than global or local methods, so they may find more use in offline analysis rather than computation *in vivo* during clinical procedures.

## Related approaches and applications

Additional properties of atrial conduction can be estimated and assessed, including conduction anisotropy, tracking activation waves, or vector field analysis. These associated analyses can be used together with CV for mechanistic assessment in the clinic and to personalize atrial models. We will briefly discuss these analyses and applications here.

### Conduction anisotropy

An extension beyond the calculation of conduction velocity is to consider the dependence of conduction speed on propagation direction, which is important to assess as atrial tissue exhibits anisotropic conduction [92, 93]. A local method for estimating anisotropy was developed by extending the generalized wave fitting approaches to consider an elliptical wave of activation, so that the fit could consider longitudinal and transverse conduction speeds, and the orientation of the longitudinal conduction direction [35]. More advanced inverse methods that also estimate conduction anisotropy were given above [83–85].

### Other methods for tracking wavefront directions

Other wavefront tracking techniques have been developed that consider wavefront propagation vectors over time. These propagation vectors can either be constructed using CV vectors calculated through LAT assignment, or through tracking constant values of another variable — for example, isopotential or isophase methods. Directed graph mapping assigns propagation vectors and uses a network theory approach to determine re-entrant wavefront activation directions [94] and determine atrial tachycardia and flutter mechanisms [95, 96]. Pathways of activation during atrial fibrillation can be assessed through optical flow mapping or Electrographic Flow mapping [97, 98], and this analysis is available in the clinic through Ablacon Ablamap Software [98].

### Clinical systems and mechanistic assessment

Conduction velocity measurements can be calculated across different pacing rates to test how the atrial tissue responds to changes in activation interval. These conduction velocity restitution curves provide information on how atrial conduction speeds are likely to change with changing activation intervals and at the shorter cycle lengths observed in atrial arrhythmias, including tachycardia and fibrillation. Weber et al. presented a pacing protocol for measuring CV restitution [99]. Conduction velocity restitution was measured in humans by Lalani et al. to demonstrate conduction slowing immediately before the onset of AF [100]. Recent tools have been developed to automate conduction velocity restitution, for example Nothstein et al. [101].

Conduction velocity vector fields can be post-processed through vector calculus to calculate properties of the wavefront propagation field, including focal activation (peaks of divergence) and rotational activity (by calculating curl) [102, 103]. Yavin et al. demonstrate a technique for using activation vectors to differentiate between conduction gap and conduction block for interrogating regions of the substrate [104]. Luther et al. highlighted the importance of carefully assigning LAT when differentiating between conduction mechanisms [105].

## Discussion

Besides direct quantitative comparison, there are three criteria we can consider for assessing different methods: (i) accounting for physics; (ii) uncertainty quantification in predictions; (iii) accounting for the manifold. Local methods can account for physics under certain simplifying assumptions about constant conduction velocity, as well as accounting for the manifold by locally flattening the mesh. Uncertainty quantification can be easily incorporated into most local methods. Global methods cannot account for physics, but can directly account for the manifold in a more general way and can incorporate uncertainty quantification in a way that is consistent with all data. This is as opposed to local methods, for which independent fits at different locations are not really self-consistent as correlations cannot be accounted for. Inverse methods approximately account for all three criteria, and future work may improve upon this even further.

How important it is to incorporate physics constraints or account for the manifold is not clear. Of the inverse methods, PINNs only weakly do both of these things whereas PIEMAP strongly does both. Local methods may be able to consider the manifold to be approximately flat, where global methods probably need to account for the manifold. The importance of uncertainty quantification in making predictions from sparse and noisy data should not be understated, especially for methods aimed at personalizing electrophysiology models that might be used for informing clinical decisions. Whereas inverse methods may be the best option for model personalization to date, in our opinion the uncertainty quantification of these techniques in particular needs much more development. Although only a few of the methods outlined in this article actually incorporated uncertainty quantification in existing publications (exceptions being [26, 65, 82]), we have tried to highlight where and how uncertainty quantification can be incorporated into existing methods.

It is difficult to calculate CV during atrial arrhythmias due to challenges in assigning LAT for complicated activation: (i) electrograms may be multi-component, low-voltage, and fractionated; (ii) there may be multiple wavefronts and wavefront collision; (iii) wavefronts may take complicated paths, including re-entry. Only local methods for CV can be applied in these complex cases. Current technologies for assessing electrical activity during atrial fibrillation, including LAT assignment during AF, have been recently reviewed [106]. In particular, the importance of detecting and removing signal noise, subtracting far-field QRS and annotating AF potentials have been highlighted. Techniques have been developed to assign LAT to complex signals including wavelet decomposition, morphological approaches, and tracking wavefronts [107–109]. Uncertainty quantification techniques for LAT assignment have also been developed [26], but the correct activation would need identifying and bracketing first prior to applying the method. In the instance of wavefront collisions, CV may be assigned to individual wavefronts if these wavefronts are first identified and analysed separately, for example using the residual of a wavefront fit to screen wavefronts [57, 58, 110]. Another approach is to mark all possible LAT on all electrograms and choose the most consistent set as an activation vector map [111]. These activation vector maps can then be analysed with physiological constraints to find areas of conduction slowing and complex circuits. Techniques have also been developed to calculate CV in the case of re-entrant circuits [59–62]. It is important to correctly assign LAT and interpret activation patterns to differentiate re-entry from pseudo-reentry [112]. During fibrillation there may be multiple wavefronts with short wavelengths so the spatial resolution of the recording device should be considered during this assessment [16].

This review article has attempted to give an overview of the different categories of methods for calculating conduction velocity. We hope our proposed categorization offers some insight, and helps expose the assumptions, limitations, and benefits of different techniques. Another reason for presenting methods in this way is that it is currently very difficult to compare methods in any other way. In our opinion, there is a clear need for benchmark data for evaluating algorithms for calculating conduction velocity. Reproducible simulated data where the ‘ground truth’ LAT (and therefore CV) is known everywhere would probably be the easiest solution, especially as computational electrophysiology simulations are extremely well developed [113, 114]. Open-source code for methods is important [23] but is only part of the solution, as its existence can imply that the burden of quantitative comparison (against an ever increasing cannon of previous methods) should be placed entirely on researchers proposing new methods, rather than being shared amongst the research community more broadly. Hopefully the next CV review paper will be a quantitative evaluation of methods by many different authors working using many different methods on the same benchmark data.

## Conclusion

Atrial conduction velocity mapping algorithms are under continuous development in research laboratories and in industry. Our aim in this review article was to give a broad overview of the different categories of currently published methods for calculating CV, and to give insight into their different advantages and disadvantages in the context of different applications. Our approach was to group techniques into local, global, and inverse methods, and discuss these techniques with respect to their compliance to the governing physics, scope to quantify uncertainty, and their ability to take into account the atrial manifold. Although further research is likely to be promising, quantitative evaluation of different techniques on a common ground truth dataset is still lacking, and hopefully future work will aim at addressing this shortcoming.

## Appendix

### A.1 Bayesian linear models

It is common to assume that noisy measurements *y* of a variable *f* have normally distributed errors $p(y | f) \sim \mathcal {N}(y | f, {\sigma _{n}^{2}} I)$. A linear model *f* = Φ***β*** can be given normal priors on the basis weights $p(\boldsymbol {\beta } | \sigma _{\beta }) \sim \mathcal {N}(\boldsymbol {\beta } | \mathbf {0}, \sigma _{\beta }^{2} I)$. The posterior distribution for the weights is then given by:

5 $$\begin{array}{@{}rcl@{}} p(\beta | x, y, \sigma_{n}, \sigma_{\beta}) \sim \mathcal{N}({\Sigma} {\Phi}^{T} \mathbf{y}, {\sigma_{n}^{2}} {\Sigma}) \end{array}$$

6 $$\begin{array}{@{}rcl@{}} {\Sigma} = \left(({\sigma_{n}^{2}} / \sigma_{\beta}^{2}) I + {\Phi}^{T}{\Phi} \right)^{-1} \end{array}$$

Values of *σ*_*β*_, *σ*_*n*_, as well as any hyperparameters in the basis functions, can be determined by maximizing the marginal (log-) likelihood. The posterior distribution of *f* follows easily from above; the posterior mean can be recognized as the expression for regularized least squares solution. It is fairly simple to include heterogeneous errors. Any methods that fit LAT using a linear model can be readily fit into a Bayesian framework in this way, and posterior samples can be used to calculate uncertainty about CV.
